# [^18^F]FDG PET/CT can trigger relevant oncological management changes leading to favorable outcome in iodine-negative thyroid cancer patients

**DOI:** 10.1007/s12020-023-03645-8

**Published:** 2023-12-22

**Authors:** Yingjun Zhi, Takahiro Higuchi, Stephan Hackenberg, Rudolf Hagen, Manuel Stöth, Agmal Scherzad, Andreas K. Buck, Rudolf A. Werner, Sebastian E. Serfling

**Affiliations:** 1 Department of Otorhinolaryngology, Plastic, Aesthetic and Reconstructive Head and Neck Surgery, University Hospital Würzburg, Würzburg, Germany; 2 Department of Nuclear Medicine, University Hospital Würzburg, Würzburg, Germany; 3 Faculty of Medicine, Dentistry and Pharmaceutical Sciences, Okayama University, Okayama, Japan; 4 Department of Otorhinolaryngology – Head and Neck Surgery, RWTH Aachen University, Aachen, Germany; 5 Johns Hopkins School of Medicine, The Russell H Morgan Department of Radiology and Radiological Science, Baltimore, MD USA

**Keywords:** [^18^F]FDG, Thyroid cancer, [^131^I], Management change, Tyrosine kinase inhibitor

## Abstract

**Background:**

In patients with iodine-negative thyroid cancer (TC), current guidelines endorse an [^18^F]FDG PET/CT to identify dedifferentiated sites of disease. We aimed to determine the rate of oncological management changes triggered by such a molecular imaging approach, along with the impact on outcome.

**Methods:**

42 consecutive patients with negative findings on [^131^I] whole body scan were scheduled for [^18^F]FDG PET/CT and treatment based on PET results were initiated. To determine the impact on oncological management, we compared the therapeutic plan prior to and after molecular imaging. Based on imaging follow-up, the rate of controlled disease (CD, defined as stable disease, complete or partial response) was also recorded, thereby allowing to assess whether [^18^F]FDG-triggered management changes can also lead to favorable outcome.

**Results:**

We observed no alterations of the treatment plan in 9/42 (21.4%) subjects (active surveillance in 9/9 [100%]). Oncological management was changed in the remaining 33/42 (78.6%; systemic treatment in 9/33 [27.3%] and non-systemic treatment in 24/33 [72.7%]). Among patients receiving non-systemic therapy, the following changes were noted: surgery in 20/24 (83.3%) and radiation therapy in 4/24 (16.7%). In the systemic group, tyrosine kinase inhibitor (TKI) was prescribed in 8/9 (88.9%), while radioiodine therapy based on a TKI-mediated redifferentiation approach was conducted in 1/9 (11.1%). In 26 subjects with available follow-up, rate of CD was 22/26 (84.6%) and among those, 15/22 (68.1%) had experienced previous management changes based on PET/CT findings.

**Conclusions:**

In subjects with iodine-negative TC, [^18^F]FDG PET/CT triggered relevant management changes along with disease control in the vast majority of patients. As such, in dedifferentiated TC, [^18^F]FDG PET/CT may serve as a relevant management tool and therapeutic decision-aid in the clinic.

## Introduction

Current guidelines endorse radioiodine therapy (RIT) after thyroidectomy in patients diagnosed with differentiated thyroid cancer (TC) and such a sequential therapeutic approach provides favorable outcome along with complete remission (CR) in most of the patients [1]. A [^131^I] whole body scan (WBS) may rule out thyroid remnants or widespread metastatic disease. Downregulation of the sodium-iodine symporter, however, may then cause a [^131^I] loss in target lesions [2], indicative for dedifferentiation of disease. This clinical scenario, however, may then pose a challenge for the treating endocrinologist or nuclear medicine specialist. As such, negative WBS then requires further diagnostic work-up to identify active sites of disease [1]. For instance, conventional imaging including computed tomography (CT) or magnetic resonance imaging (MRI) have yielded mixed results in detecting dedifferentiating lesions [3]. [^18^F]FDG, however, has also been applied in such patients and has provided excellent read-out capabilities due to increased glucose consumption in TC lesions with [^131^I] loss [4]. In this regard, a growing body of evidence has already demonstrated the clinical value of a dual-radiotracer strategy of [^131^I] WBS / [^18^F]FDG PET/CT. Among others, Palmedo et al. reported on a diagnostic accuracy of more than 90% in this patient population [4], while quantitative parameters derived from [^18^F]FDG also had prognostic ability for outcome [5, 6]. Given those favorable diagnostic and prognostic performance, a recent study investigated the impact of [^18^F]FDG on oncological management in iodine-negative TC patients and reported on 10/21 (48%) with altered treatment plan [4]. However, in this previous investigation, the therapeutic armamentarium in dedifferentiated disease did not include tyrosine kinase inhibitor (TKI) or redifferentiation approaches for re-administration of [^131^I] at that time. Thus, in this previous study, the authors only reported on surgical intervention as management changes, i.e., alterations of the treatment plan were limited to non-systemic therapeutic changes [4].

In the present study, we aimed to determine the impact on oncological management of [^18^F]FDG PET/CT in iodione-negative TC patients, particularly focusing on systemic treatment alterations (TKI, RIT after iodine uptake restoration) vs. non-systemic changes (surgery, external beam radiation [RTx]).

## Material and methods

### General

Due to the retrospective character, the local ethics committee at the University Hospital of Würzburg waived the need for further approval (No. 20230721 01). A previous case reported on one subject of this cohort which was scheduled for a redifferentiation approach, i.e., RIT was performed due to restored radioiodine uptake mediated by previous TKI intake [7].

### Patient population

42 patients (29 female) between 18 and 85 years (59.7 ± 19.4) with a histologically confirmed TC (27/42 [64.3%] papillary, 11/42 [26.2%] follicular, 3/42 [7.1%] oncocytic, and 1/42 [2.4%] insular TC) were included. All patients had undergone previous surgery and RIT (median cumulative activity, 12 GBq [^131^I]) and showed no relevant uptake on WBS along with increasing tumor marker thyroglobulin (TG). [^18^F]FDG PET/CT for restaging was then performed as part of clinical routine follow-up. All subjects included in the study gave informed consent for procedures. A supplementary table provides a detailed overview on previous therapies and TG at time of scan.

### Imaging procedures

[^18^F]FDG PET/CT low dose technique was performed in all patients with a Siemens Biograph mCT 64 or 128 (Siemens Healthineers, Erlangen, Germany) scanner. The imaging procedure was performed 60 min after the injection of approximately 292 ± 41 MBq of [^18^F]FDG. A scan of the whole body (from the top of the skull to the proximal thigh) was performed. The section thickness of the PET/CT was 5 mm. All PET images were iteratively reconstructed in accordance with the manufacturer’s instructions (Siemens Healthineers, Erlangen, Germany).

### Change in oncological management based on [^18^F]FDG PET/CT

Treatment plans right before and after [^18^F]FDG PET/CT were retrieved from our medical archive. Treatment alterations were either classified as systemic (TKI [including dose alterations], redifferentation approach using RIT after TKI intake) or non-systemic therapy (surgery, RTx). Surgical approaches were also divided into cervical vs. thoracic procedures.

### Impact of management change on disease control

To determine the impact of management change on CD, the first follow-up imaging (conducted within one year) after [^18^F]FDG PET/CT was assessed. We applied RECIST criteria 1.1. Stable, partial, or complete response was then summarized as CD [8, 9] .

### Statistical analysis

Descriptive statistics are indicated as mean ± SD. We used Excel for Windows 2016 (Microsoft, Redmond, WA, USA) and GraphPad Prism Software (9.3.1; La Jolla, CA, USA).

## Results

### [^18^F]FDG PET/CT identified sites of disease in all patients

On a patient-based level, [^18^F]FDG PET/CT was positive in all subjects (100%) and the following disease sites on an organ-based level were recorded: 9/42 (21.4%) patients had a local recurrence, and 24/42 (57.1%) presented with lymph node (LN) metastases [16/24 (66.7%) cervical and 8/24 (33.3%) mediastinal LN]. 21/42 (50%) had lung, 4/42 (9.5%) bone and 1/42 (2.4%) liver metastases (Table 1).

**Table 1:** Patient’s characteristics

Patient #	Previous surgery (thyroidectomy) n/0, y/1	Previous locoregional (neck) surgery n/0 y/1	Overall number of previous surgeries	Number of RIT	Cumulative activity in GBq I-131	Previous TKI n/0, y/1	Duration of TKI therapy (months)	TG at time of scan	Change of management (0/1)	[^18^F]FDG PET: LR/DM findings attributable to change in management
**1**	1	1	2	1	4	0	0	4	1	LN, DM P
**2**	1	0	1	1	4	0	0	17	1	LN, DM P
**3**	1	0	1	3	18	0	0	4.2	1	DM P
**4**	1	0	1	1	4	0	0	1	1	LN, DM P
**5**	1	1	2	7	46	0	0	910	1	LN
**6**	1	0	1	1	4	0	0	10.9	1	LN, DM P
**7**	1	0	1	4	24	0	0	2010	1	LN, DM P
**8**	1	1	2	4	24	0	0	126	1	LN, DM P
**9**	1	0	1	2	11	0	0	0.4	1	LR
**10**	1	1	2	3	18	0	0	3.5	1	LN
**11**	1	0	1	2	10	0	0	0.4	1	LN
**12**	1	0	1	3	17	0	0	1.2	1	LN, DM P
**13**	1	0	1	2	11	0	0	43.5	1	LR
**14**	1	0	1	3	18	0	0	187	1	DM P
**15**	1	1	2	3	18	0	0	1152	1	DM B
**16**	1	1	2	2	13	0	0	15	1	LN, DM B
**17**	1	0	1	2	10	0	0	0.2	1	LN
**18**	1	0	1	1	4	0	0	304	1	DM P
**19**	1	0	1	2	11	0	0	19	1	LN, DM P
**20**	1	0	1	3	18	0	0	76	1	LN
**21**	1	0	1	2	11	0	0	0.2	1	DM P
**22**	1	0	1	5	32	0	0	43	1	LR, DM P
**23**	1	1	2	3	16	0	0	60	1	LN, DM P
**24**	1	0	1	2	10	0	0	142	1	DM P
**25**	1	1	2	3	18	0	0	70	1	DM P
**26**	1	0	1	1	4	0	0	31	1	LR, LN
**27**	1	0	1	1	4	0	0	215	1	LR, LN, DM B
**28**	1	1	2	3	16	0	0	5.4	1	LN, DM P
**29**	1	1	2	4	25	1	84	233	1	LR
**30**	1	0	1	3	18	1	120	7	1	LR, DM L+B
**31**	1	0	1	1	4	0	0	16	1	LN
**32**	1	1	2	2	11	0	0	34	1	LN
**33**	1	1	2	2	11	0	0	143	1	LR, LN
**34**	1	1	2	2	11	0	0	87	0	LN
**35**	1	0	1	4	25	0	0	0.4	0	LR
**36**	1	0	1	3	18	0	0	17	0	DM P
**37**	1	0	1	2	10	0	0	11	0	DM P
**38**	1	0	1	4	21	0	0	410	0	DM P
**39**	1	0	1	2	11	0	0	0	0	LN
**40**	1	1	2	2	13	0	0	0.3	0	LN
**41**	1	1	2	4	25	0	0	86	0	DM P
**42**	1	1	2	1	4	0	0	0	0	-

*RIT* radioiodine therapy, *GBq* Gigabecquerel, *TKI* tyrosine kinase inhibitors, *TG* thyroglobulin, *DM* distant metastases, *LR* local recurrence, *LN* lymph node, *P* pulmonal, *B* bone, *L* liver

### [^18^F]FDG PET/CT led to change in oncological management in more than 78%

We observed no changes of the treatment plan after [^18^F]FDG PET/CT in 9/42 (21.4%) subjects and in those individuals, active surveillance was conducted (9/9 [100%]).

**Fig. 1:**
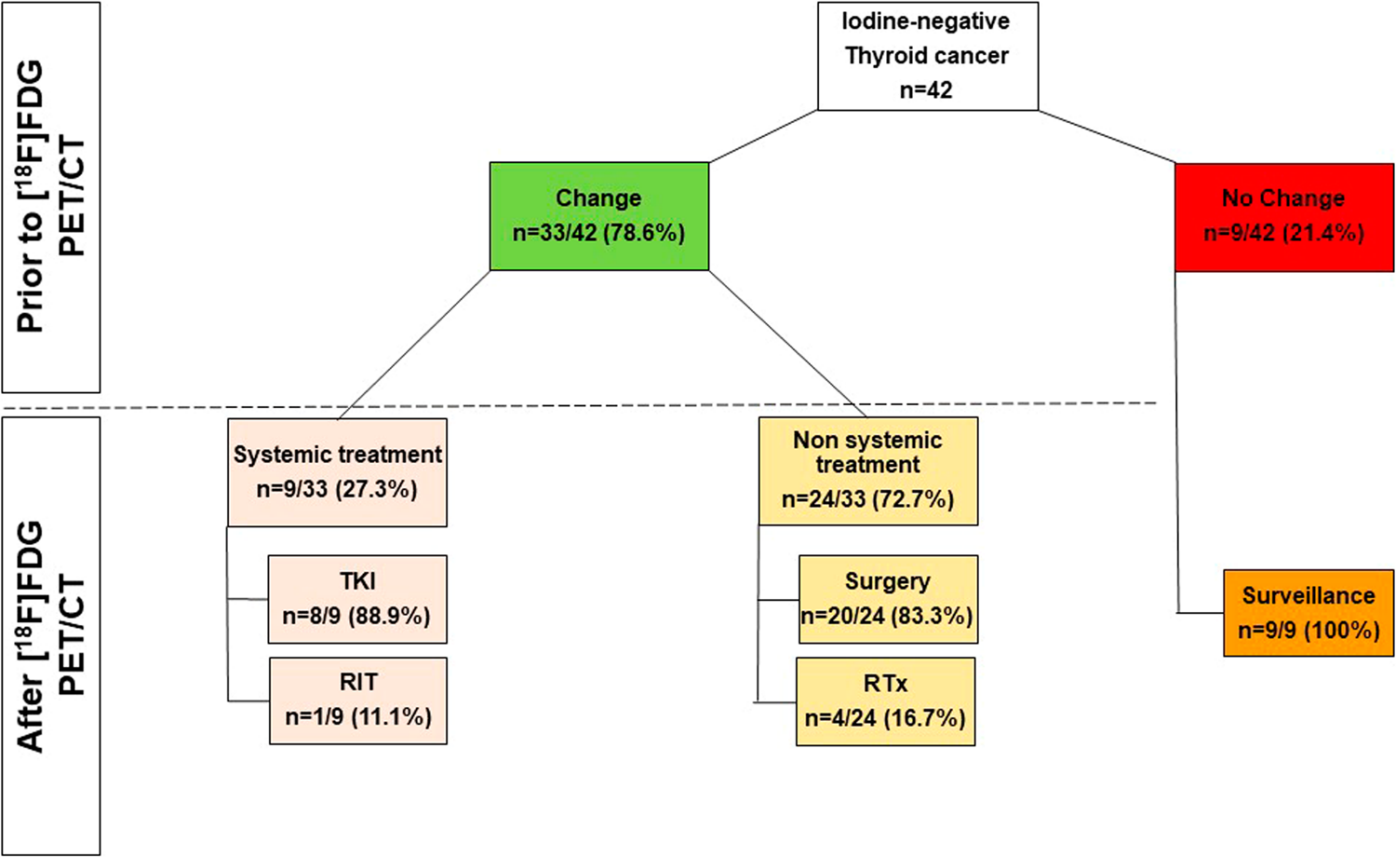
Decision tree of patients scheduled for [^18^F]FDG PET/CT after negative whole-body scan. In more than 78%, PET/CT triggered management changes, with the vast majority allocated to the non-systemic group

**Fig. 2:**
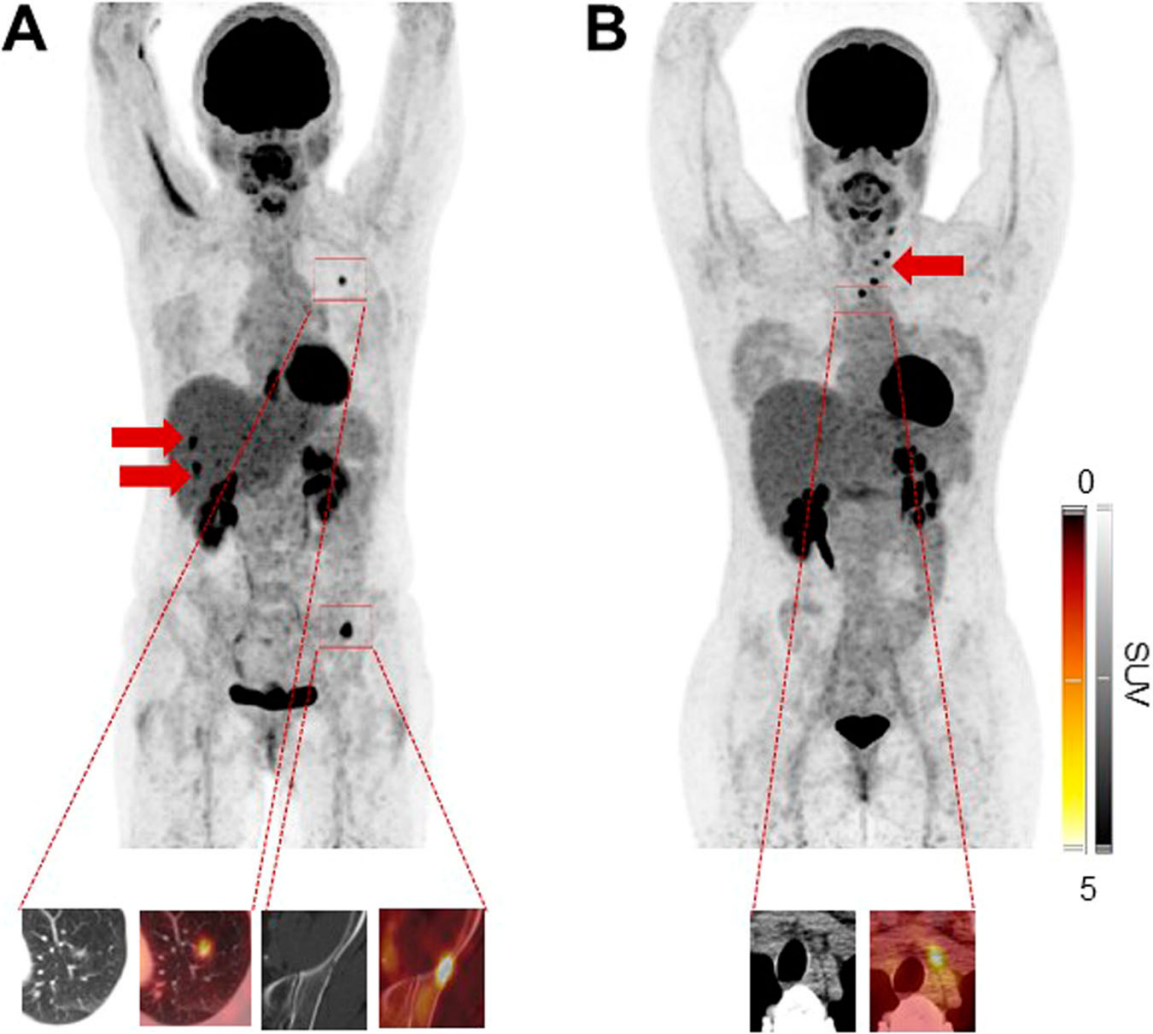
Patients with management changes after [^18^F]FDG PET/CT, with (**A**) systemic and (**B**) non-systemic treatment alterations. Maximum intensity projections and transaxial CT and PET/CT portions of target lesions are also displayed. Subject in **A** showed multiple organ involvement with PET-positive pulmonal and osseous findings (in the left pelvis) displayed on transaxial CT and PET/CT. Of note, lung lesions were only identifiable in the PET/CT images. Additional PET-positive hepatic lesions are not shown on transaxial slides, but seen on the MIP (arrows). Given widespread disease revealed by [^18^F]FDG, this patient was scheduled for tyrosine kinase inhibitor. Patient in **B**, however, only presented with cervical PET-positive lymph nodes (arrow, also seen on transaxial PET and PET/CT), triggering neck dissection of [^18^F]FDG-avid findings. Both patients then presented with controlled disease upon follow-up

Oncological management was changed in the remaining 33/42 (78.6%). When subdividing those patients into non-systemic vs. systemic therapy, 9/33 (27.3%) were allocated to the latter subgroup. TKI was then prescribed in 8/9 (88.9%), while radioiodine therapy was conducted in 1/9 (11.1%, applying a TKI-based redifferentiation approach). After [^18^F]FDG, non-systemic treatment was then applied in 24/33 (72.7%) and those included RTx in 4/24 (16.7%) and surgery in 20/24 (83.3%; Fig. 1). Among patients scheduled for operative procedures, cervical surgery was conducted in 12/20 (60.0%) and thoracic surgery in 8/20 (40.0%). Figure 2 provides two cases, in whom [^18^F]FDG PET/CT led to systemic treatment initiation (TKI, **A**) and non-systemic management change (cervical LN dissection, **B**).

### [^18^F]FDG PET/CT-triggered management changes led to disease control in more than 68%

In the entire cohort, follow-up imaging was available in 26/42 (61.9%) and CD was then recorded in 22/26 (84.6%). In this subgroup, 15/22 (68.1%) had experienced previous management changes based on PET findings and in those subjects, the majority (12/15 [80%]) had received non-systemic treatment (surgery in 9/12 [75%] and RTx in 3/12 [25%]). The remaining 3/12 (25%) presenting with CD upon follow-up, however, had received systemic treatment after PET/CT (TKI in 2/3 [66.7%] and RIT in 1/3 [33.3%]). In a supplementary table, we provided a comparison of the pathological findings on cross sectional imaging relative to [^18^F]FDG PET, which led to respective management changes.

## Discussion

In patients with dedifferentiated TC scheduled for [^18^F]FDG after the first negative WBS, PET/CT led to management changes in more than 78% of the patients. Of those subjects, the majority was allocated to non-systemic alterations, mainly including surgical interventions. After PET/CT, however, approximately one-fourth of the subjects were also treated systemically using TKI (or TKI in combination with RIT). Of note, the [^18^F]FDG-triggered change in management then also led to disease control in more than 68% of the subjects. Taken together, even with an increasing therapeutic armamentarium in iodine-negative TC patients [10], [^18^F]FDG PET/CT provided crucial therapeutic decision-aid to identify the most appropriate treatment. Of note, this applied to subjects with limited (scheduled for surgery or RTx) and patients with widespread disease (triggering TKI prescription or TKI-mediated RIT).

Loss of sodium-iodine transporter in TC may pose a challenge, as RIT no longer provides anti-tumor efficacy and thus, in patients with negative WBS with or without rising TG, [^18^F]FDG may identify dedifferentiating tumor lesions [4]. Previous reports have reported on excellent diagnostic accuracy [4], but information on clinical relevance is rather limited. In this regard, a previous investigation published in 2006 showed that PET/CT can change management in a substantial fraction of patients, mainly surgical interventions. In the last two decades, a plethora of novel therapies have entered the clinical arena [10], including lenvatinib in iodine-refractory TC with response rates of almost 65% when compared to placebo [11]. Moreover, favorable outcome has also been reported using pazopanib or sorafenib [12, 13], which even led to approval by respective agencies [14]. As such, given the beneficial evidence of such oral kinase inhibitors in iodine-refractory subjects in recent years, we aimed to determine whether [^18^F]FDG is still useful to guide the treating physician towards the most appropriate next therapeutic step beyond surgical procedures. We recorded management alterations in >78% of the subjects, with disease control in 68.1% that had experienced previous changes of the treatment plan. This is slightly increased when compared to the findings of Palmedo et al. (48%) [4] and may be partially explained by the improved scanner technology in our study (Biograph mCT 64 or 128) along with improved read-out. Systemic treatment alterations were rather less frequent in our analysis and included use of TKI, but also redifferentiation approaches, i.e., restoration of radioiodine uptake due to TKI-mediated modulation of the sodium-iodine symporter in TC cells [15]. Due to the increasing use of such sophisticated therapeutic approaches of target re-activation [7, 16], our study also provides a hint that [^18^F]FDG could serve as a decision-aid to identify patients eligible for such a salvage approach.

Of note, comparable to previous work [4], we also recorded surgery as the most frequent intervention after [^18^F]FDG and those findings are most likely due to the fact that patients were included right after the first negative WBS (i.e., at an early stage of re-differentiating disease). As such, future studies may also investigate individuals at later stage, which may then also demonstrate that [^18^F]FDG PET/CT can also trigger a higher rate of systemic therapeutic interventions (such as TKI). Nonetheless, the present and previous studies indicate that this radiotracer is beneficial in subjects presenting with iodine-negative WBS. This applies in particular to limited cervical disease, where [^18^F]FDG can then precisely localize metastatic LN, thereby guiding the endocrine surgeon or otorhinolaryngologist in pre-operative planning. Of note, a substantial fraction of individuals in our study also presented with thoracic findings revealed by molecular imaging, which then also triggered interventions by thoracic surgery in 40% (including dissection of mediastinal LN and lung metastases). Those findings may be of relevance, as a previous study also reported that loss of radioiodine avidity in pulmonal lesions is an independent predictor of poor outcome, thereby emphasizing the importance of an accurate thoracic read-out by [^18^F]FDG PET/CT in those high-risk patients [17].

Prospective studies are needed to corroborate our preliminary findings. Future analyses, however, may also consider to repeat our investigation in a larger number of patients, by pooling data from multiple centers or by using other, sensitive PET agents, e.g., [^18^F]tetrafluoroborate [18, 19] or theranostic agents also used in such a patient population, e.g., prostate-specific membrane antigen-targeted PET/CT [20].

## Conclusions

In patients with dedifferentiated TC scheduled for [^18^F]FDG after the first negative WBS, PET/CT led to management changes in the vast majority of patients, including systemic (TKI, RIT after re-differentiation) and non-systemic treatment changes (thoracic/cervical surgery, RTx). Those alterations of the treatment plan achieved controlled disease in more than 68% of the patients. The high rate of PET/CT-triggered surgical interventions indicate that [^18^F]FDG may be particularly useful to precisely localize limited cervical or widespread thoracic disease, thereby also serving as a valuable pre-operative planning tool.

### Supplementary Information

Supplementary table
