# Supplementary material for: [18F]FDG PET/CT can trigger relevant oncological management changes leading to favorable outcome in iodine-negative thyroid cancer patients
Source: Endocrine. 2023 Dec 22;84(2):656–62. doi: 10.1007/s12020-023-03645-8 (PMC11076315; doi:10.1007/s12020-023-03645-8)
Supplement: Supplementary file 1 — Supplementary table [file 12020_2023_3645_MOESM1_ESM.docx]

| **Patient #** | **Cross sectional imaging: pathological findings** | **[^18^F]FDG PET: additional pathological findings** |
| --- | --- | --- |
| **1** | pulmonal metastases | lymphonodal metastases |
| **2** | pulmonal metastases | lymphonodal metastases |
| **3** | pulmonal metastases | more pulmonal metastases |
| **4** | pulmonal metastases | lymphonodal metastases |
| **5** | lymphonodal metastases | more lymphonodal metastases |
| **6** | pulmonal metastases | lymphonodal metastases |
| **7** | pulmonal metastases + single lymphonodal metastasis | more lymphonodal metastases |
| **8** | pulmonal, lymphonodal metastases | more lymphonodal metastases |
| **9** | no pathological finding | local recurrence |
| **10** | no pathological finding | single lymphonodal metastasis |
| **11** | no pathological finding | single lymphonodal metastasis |
| **12** | no pathological finding | lymphonodal metastases, pulmonal metastases |
| **13** | no pathological finding | local recurrence |
| **14** | no pathological finding | single pulmonal metastasis |
| **15** | single bone metastasis | more bone metastases |
| **16** | lymphonodal metastases | more lymphonodal metastases, single bone metastasis |
| **17** | no pathologic finding | single lymphonodal metastasis |
| **18** | pulmonal metastases | more pulmonal metases |
| **19** | pulmonal metastases | lymphonodal metastases |
| **20** | lymphonodal metastases | more lymphonodal metastases |
| **21** | single pulmonal metastasis | more pulmonal metastases |
| **22** | pulmonal metastases | local recurrence |
| **23** | pulmonal metastases | lymphonodal metastases |
| **24** | pulmonal metastases | more pulmonal metastases |
| **25** | pulmonal metastases | more pulmonal metastases |
| **26** | lymphonodal metastases | local recurrence |
| **27** | lymphonodal metastases | local recurrence, single bone metastasis |
| **28** | pulmonal metastases | lymphonodal metastases |
| **29** | no pathological finding | local recurrence |
| **30** | pulmonal metastases | local recurrence, bone metastases |
| **31** | single lymphonodal metastasis | more lymphonodal metastases |
| **32** | lymphonodal metastases | more lymphonodal metastases |
| **33** | lymphonodal metastases | local recurrence |
| **34** | lymphonodal metastases | no additional pathologic findings |
| **35** | local recurrence | no additional pathologic findings |
| **36** | pulmonal metastases | no additional pathologic findings |
| **37** | pulmonal metastases | no additional pathologic findings |
| **38** | pulmonal metastases | no additional pathologic findings |
| **39** | lymphonodal metastases | no additional pathologic findings |
| **40** | lymphonodal metastases | no additional pathologic findings |
| **41** | pulmonal metastases | no additional pathologic findings |
| **42** | no pathologic findings | no additional pathologic findings |

**Supplementary Table.** Comparison of pathological findings on conventional imaging relative

to [^18^F]FDG PET. Cross sectional imaging includes CT and/or MRI.
